# Magnetic-Field-Enhanced Microwave Absorption of Superparamagnetic Fe_3_O_4_/RGO Composites

**DOI:** 10.3390/mi17060754

**Published:** 2026-06-22

**Authors:** Guijiang Liu, Xingbao Lyu, Yiqun Ma, Chengxun Yuan, Zhongxiang Zhou

**Affiliations:** 1 School of Physics, Harbin Institute of Technology, Harbin 150001, China; 24b311021@stu.hit.edu.cn (G.L.); myq@stu.hit.edu.cn (Y.M.); yuancx@hit.edu.cn (C.Y.); zhouzx@hit.edu.cn (Z.Z.); 2 Heilongjiang Provincial Key Laboratory of Plasma Physics and Application Technology, Harbin 150001, China; 3 Heilongjiang Provincial Innovation Research Center for Plasma Physics and Application Technology, Harbin 150001, China

**Keywords:** magnetic-field-induced alignment, microwave absorption, Fe_3_O_4_ nanoparticles, superparamagnetic relaxation

## Abstract

Superparamagnetic materials have attracted increasing attention for high-frequency microwave absorption because superparamagnetic relaxation can partially overcome the high-frequency limitations of conventional magnetic absorbers. Herein, Fe_3_O_4_/rGO composite powders were prepared by electrostatic self-assembly and subsequently incorporated into an epoxy matrix, and magnetic-field-induced alignment was introduced during curing. Owing to the synergistic effects of interfacial polarization, magnetic dissipation, and improved impedance matching, the optimized composites exhibited markedly enhanced microwave absorption performance. In particular, when the rGO content was 10 wt% and an external magnetic field was applied, the composite achieved effective absorption across the entire X-band (8–12 GHz) within a thickness range of 1–3 mm, together with a minimum reflection loss of −40.3 dB. The enhanced performance is attributed to the combined contributions of abundant heterogeneous interfaces, superparamagnetic relaxation, and field-induced orientation of Fe_3_O_4_-decorated rGO sheets. This work provides a simple physical strategy for the microstructural regulation of magnetic–dielectric composites toward high-performance microwave absorption.

## 1. Introduction

The rapid advancement of electronic communication technology, while fostering significant innovation and convenience, has inevitably led to severe electromagnetic (EM) radiation pollution [1,2,3,4,5]. This issue poses serious risks to the stable operation of precision equipment and human health. Microwave absorption materials (MAMs), which integrate superior impedance matching with robust attenuation capabilities, have emerged as an effective solution to mitigate EM pollution [6,7,8]. However, most single-phase materials struggle to simultaneously achieve optimal impedance matching and high attenuation due to their intrinsic limitations. On one hand, single-component carbon materials—ranging from 0D carbon quantum dots and 1D carbon nanotubes to 2D graphene and 3D carbon foams—are often restricted by poor impedance matching and limited EM wave attenuation pathways [9,10,11,12,13]. On the other hand, magnetic materials are constrained by high density and the Snoek’s limit at high frequencies [14,15]. Consequently, there is an urgent need to explore strategies that effectively synergize the advantages of both components to develop low-density, high-efficiency MAMs.

To introduce additional attenuation mechanisms and optimize impedance matching, various strategies have been proposed, among which the utilization of interfaces to enhance interfacial polarization is considered a highly attractive approach [16,17]. By incorporating magnetic phases such as Co, Ni, or ${\mathrm{Fe}}_{3}O_{4}$ into carbon-based substrates (e.g., graphene, porous carbon, or carbon fibers), researchers have constructed heterogeneous magnetic-carbon interfaces [17,18,19,20]. These interfaces promote space charge accumulation and interfacial polarization loss, which, in synergy with magnetic loss, significantly improve both impedance matching and EM attenuation capacity [21]. For example, Co nanoparticle-decorated carbon nanofibers with abundant structural defects, hierarchical ${\mathrm{Co}}_{2}P$/$\mathrm{Co}S_{2}$@C@$\mathrm{Mo}S_{2}$ composites with multiple heterointerfaces, and ion-doped hollow spinel absorbers have exhibited excellent microwave absorption performance through the synergistic regulation of conductive loss, interfacial polarization, magnetic loss, and impedance matching [22,23,24].

In recent years, superparamagnetic nanomaterials have garnered widespread attention due to their efficient microwave absorption at high frequencies. Superparamagnetic relaxation effectively overcomes Snoek’s limit, demonstrating impressive absorption performance at high microwave frequencies. For instance, Song and Zhang et al. synthesized $\mathrm{Si}O_{2}$-coated superparamagnetic ${\mathrm{Fe}}_{3}O_{4}$ nanoparticles, achieving relaxation frequencies of 6.9 GHz and 9.3 GHz, respectively, through superparamagnetic coupling [14,15]. Yuan et al. anchored superparamagnetic ${\mathrm{Fe}}_{3}O_{4}$ nanoparticles onto carbon nanotubes using a co-precipitation method, reaching a resonance frequency of 9.1 GHz [25]. Similarly, Anh et al. fabricated superparamagnetic ${\mathrm{Zn}}_{0.8}{\mathrm{Ni}}_{0.2}{\mathrm{Fe}}_{2}O_{4}$ alloys that exhibited an absorption attenuation exceeding 20 dB at 10 GHz [26].

Under an external magnetic field, superparamagnetic nanoparticles align into chain-like structures along the field direction through magnetic dipole interactions [27]. This magnetically aligned chain-like structure provides a more efficient pathway for microwave propagation and dissipation within the absorber and is considered a reliable strategy for regulating the microwave response of magnetic fluids [28,29]. Polymer-based electromagnetic functional materials have attracted considerable attention in electromagnetic interference shielding, microwave absorption, and multifunctional electromagnetic devices owing to their low density, processability, and structural designability [30]. If a magnetic field is applied during the polymerization process, the alignment remains embedded within the composite matrix once the solvent solidifies and the field is removed. This processing technique has already been applied to enhance mechanical properties and achieve flexible robotic manipulation [31,32,33]. Meanwhile, electrostatic self-assembly is regarded as an effective strategy for inducing microstructural organization during polymer processing [34].

In this study, a facile, low-cost, and environmentally friendly method was developed to fabricate superparamagnetic ${\mathrm{Fe}}_{3}O_{4}$/rGO composites. The self-assembly of superparamagnetic ${\mathrm{Fe}}_{3}O_{4}$ and reduced graphene oxide (rGO) was achieved via electrostatic adsorption, leveraging the potential difference between the materials. When incorporated into an epoxy resin matrix under an external magnetic field, the rGO sheets decorated with superparamagnetic iron oxide nanoparticles (SPIONs) aligned along the field lines. By combining electrostatic assembly with magnetic-field-induced structural control, the ${\mathrm{Fe}}_{3}O_{4}$/rGO composites achieved an effective absorption bandwidth (EAB) covering the entire X-band (8–12 GHz) within a thickness range of 1–3 mm, with a minimum reflection loss (${\mathrm{RL}}_{\min}$) of −40.3 dB. This work demonstrates a physical approach for microstructural reassembly of nanoscale composites via electrostatic adsorption and magnetic-field induction, providing novel insights for the development of EM shielding, microwave absorption materials, and broader composite engineering.

## 2. Materials and Methods

### 2.1. Materials

The ${\mathrm{Fe}}_{3}O_{4}$ nanoparticles (spherical morphology, 10–20 nm in diameter) were purchased from Qinghe Xingye Metal Materials Co., Ltd. (Jinan, China). Monolayer rGO was obtained from Shenzhen Suiheng Technology Co., Ltd. (Shenzhen, China). E51 epoxy resin was supplied by Kunshan Jiulimei Electronic Materials Co., Ltd. (Kunshan, China). Standard hydrochloric acid (HCl) solution and curing agents, including diethylenetriamine (DETA) and dibutyl phthalate (DBP), were provided by Sinopharm Chemical Reagent Co., Ltd. (Shanghai, China). All reagents were used as received without further purification.

### 2.2. Preparation of Composite Materials

The ${\mathrm{Fe}}_{3}O_{4}$/rGO composite powders were synthesized via an electrostatic adsorption method. Typically, specified amounts of ${\mathrm{Fe}}_{3}O_{4}$ and rGO powders were dispersed separately in deionized (DI) water. After stirring for 5 min, the mixtures were subjected to pulsed ultrasonication for 30 min to obtain 0.1 g/L dispersions of ${\mathrm{Fe}}_{3}O_{4}$ and rGO, respectively. Subsequently, the pH values of the two dispersions were adjusted to 5.5–6.0 by adding a standard hydrochloric acid solution. As shown in Figure S1, at this pH, the zeta potential of the ${\mathrm{Fe}}_{3}O_{4}$ nanoparticles was approximately +10 mV, whereas that of rGO was approximately −8 mV. The opposite signs of their surface charges enabled electrostatic-attraction-driven self-assembly between the ${\mathrm{Fe}}_{3}O_{4}$ nanoparticles and the rGO sheets, resulting in the immobilization of ${\mathrm{Fe}}_{3}O_{4}$ nanoparticles on the rGO surface. The two dispersions were then subjected to an additional 2 min of ultrasonication. Under constant magnetic stirring at 500 rpm, the rGO dispersion was added dropwise into the ${\mathrm{Fe}}_{3}O_{4}$ dispersion, with the volume of the rGO dispersion controlled according to the mass ratios listed in Table 1. The resulting mixture was briefly ultrasonicated for 2 min and then stirred continuously at room temperature for 2 h to promote self-assembly. The ${\mathrm{Fe}}_{3}O_{4}$/rGO composite precipitate was collected through magnetic separation, and the supernatant was discarded. The obtained precipitate was washed twice with DI water, involving 1 min of ultrasonication followed by magnetic separation for each cycle. Finally, the composites were dried in a drying oven at 50 °C for 12 h to obtain ${\mathrm{Fe}}_{3}O_{4}$/rGO composite powders.

**Figure 1:**
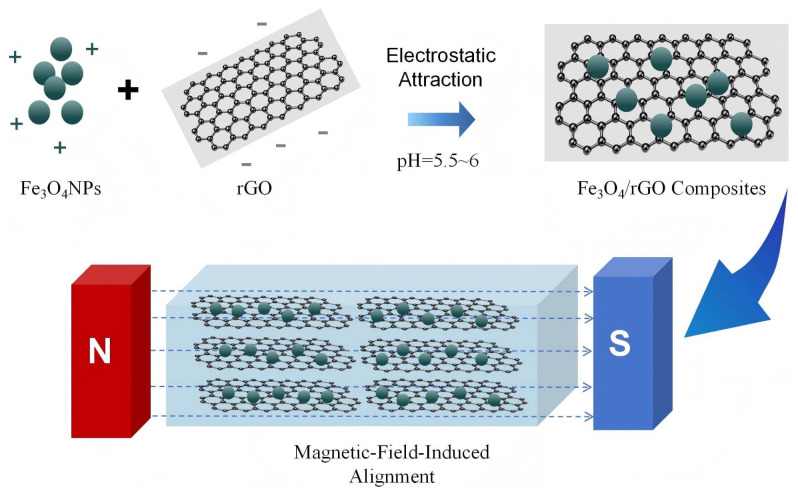
Schematic illustration of the material preparation process. The green spheres, gray sheets, and red/blue blocks represent Fe_3_O_4_ nanoparticles, rGO nanosheets, and the N/S poles of permanent magnets, respectively. The ’+’ and ’−’ symbols denote opposite surface charges.

The fabrication process of the ${\mathrm{Fe}}_{3}O_{4}$/rGO/epoxy composites is described as follows: (1) Premixing: The as-prepared ${\mathrm{Fe}}_{3}O_{4}$/rGO composite powders were mixed with epoxy resin according to the weight ratios specified in Table 1. The mixture was subjected to mechanical stirring at 500 rpm for 30 min at room temperature to ensure uniform dispersion. (2) Curing-agent Addition and Degassing: Subsequently, the curing-agent was added to the mixture at a resin-to-curing agent weight ratio of 5:1 and stirred for an additional 15 min. The resulting mixture was then allowed to stand for 10 min to remove entrapped air bubbles. (3) Casting and Magnetic Alignment: The mixture was quickly cast into customized silicone waveguide molds (fabricated via 3D printing/additive manufacturing). The internal dimensions of the molds were 22.86 × 10.16 × 2 mm (as shown in Figure S2a), corresponding to standard waveguide requirements. To induce directional alignment, the molds were placed between two square permanent magnets (50 × 50 × 20 mm). The magnetic field intensity at the sample center was measured to be 80 mT using a Gauss meter (DLX-STW1044), as illustrated in Figure S2b. (4) Curing: The samples were cured at room temperature for 6 h under the applied magnetic field. For comparison, a control group was prepared under identical conditions but without the external magnetic field. Three types of ${\mathrm{Fe}}_{3}O_{4}$/rGO composites with different mass fractions were fabricated (see Table 1). The overall preparation workflow is schematically illustrated in Figure 1.

### 2.3. Characterization

The phase compositions of the ${\mathrm{Fe}}_{3}O_{4}$ nanoparticles and ${\mathrm{Fe}}_{3}O_{4}$/rGO composites were analyzed by X-ray diffraction (XRD, PANalytical X’Pert PRO). The zeta potentials of the ${\mathrm{Fe}}_{3}O_{4}$ and rGO powders were measured using a Zeta potential analyzer (Zeta potential, Malvern Zetasizer Nano ZSP) to determine their surface charge states. The microstructure and surface morphology of the ${\mathrm{Fe}}_{3}O_{4}$ nanoparticles and the composites were characterized via scanning electron microscopy (SEM, Zeiss Gemini 560). Magnetic properties, including hysteresis loops, were investigated using a vibrating sample magnetometer (VSM, Lake Shore 7404). The electromagnetic (EM) parameters of the samples were measured in the X-band (8–12 GHz) using a vector network analyzer (VNA, Keysight PNA-L) via the waveguide method. Before testing, the VNA was calibrated using a 32118 waveguide calibration kit.

## 3. Results and Discussion

### 3.1. Composition and Structure of Composite Materials

**Figure 2:**
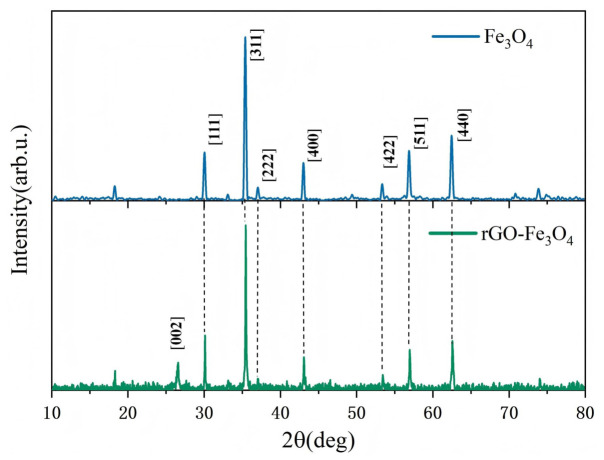
X-ray diffraction patterns of ${\mathrm{Fe}}_{3}O_{4}$ nanoparticles and ${\mathrm{Fe}}_{3}O_{4}$/rGO powder.

Figure 2 shows the XRD patterns of the ${\mathrm{Fe}}_{3}O_{4}$ nanoparticles and ${\mathrm{Fe}}_{3}O_{4}$/rGO composites. The diffraction peaks of ${\mathrm{Fe}}_{3}O_{4}$ are indexed to the (111), (311), (222), (400), (422), (511), and (440) planes, which are in good agreement with the standard cubic spinel phase (JCPDS No. 19-0629). The sharp and intense peaks indicate a high degree of crystallinity in the ${\mathrm{Fe}}_{3}O_{4}$ NPs. No characteristic peaks of impurities or intermediate phases were detected, confirming the high chemical and phase purity of the nanoparticles [35]. For the ${\mathrm{Fe}}_{3}O_{4}$/rGO composites, the characteristic peaks of ${\mathrm{Fe}}_{3}O_{4}$ remain prominent, while the appearance of the (002) plane confirms the successful incorporation of rGO [36].

**Figure 3:**
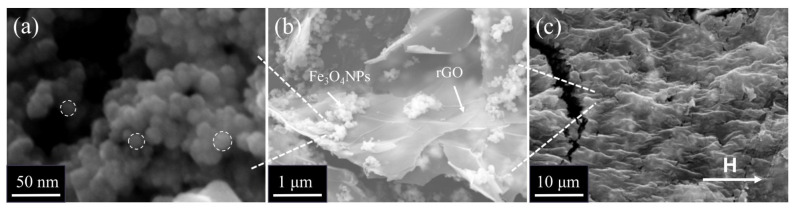
SEM image of ${\mathrm{Fe}}_{3}O_{4}$/rGO composites: (**a**) ${\mathrm{Fe}}_{3}O_{4}$ nanoparticles, (**b**) ${\mathrm{Fe}}_{3}O_{4}$ nanoparticles decorated on rGO sheets, and (**c**) field-induced alignment of ${\mathrm{Fe}}_{3}O_{4}$/rGO sheets in the epoxy matrix. The circles indicate the outlines of individual Fe_3_O_4_ nanoparticles, and the dashed lines indicate the regions in the preceding image that correspond to the subsequent enlarged image.

As illustrated in Figure 3a, the particle size of the ${\mathrm{Fe}}_{3}O_{4}$ NPs is primarily distributed between 10 and 20 nm. Since this size range is below the critical threshold for superparamagnetism in ${\mathrm{Fe}}_{3}O_{4}$ (typically 25 nm), the nanoparticles are expected to exhibit superparamagnetic behavior [37,38]. Considering the particle size distribution of the ${\mathrm{Fe}}_{3}O_{4}$ nanoparticles during sample preparation, a small fraction of the nanoparticles may form small clusters, whereas the majority of the nanoparticles satisfy the size criterion for superparamagnetic behavior. The SEM images reveal that the rGO maintains a single-layer, sheet-like structure. Through electrostatic adsorption, the ${\mathrm{Fe}}_{3}O_{4}$ NPs clusters are effectively decorated onto the surface of the rGO nanosheets. Furthermore, as shown in Figure 3c, when an external magnetic field is applied, the ${\mathrm{Fe}}_{3}O_{4}$-decorated rGO sheets exhibit a clear directional alignment along the magnetic field lines. This can be attributed to the fact that, under the influence of the external magnetic field, the nonzero average magnetic moments generated by the superparamagnetic ${\mathrm{Fe}}_{3}O_{4}$ nanoparticles tend to align along the direction of the applied field, thereby driving the orientation of the ${\mathrm{Fe}}_{3}O_{4}$/rGO composite sheets within the epoxy matrix.

### 3.2. Magnetic Characterization

**Figure 4:**
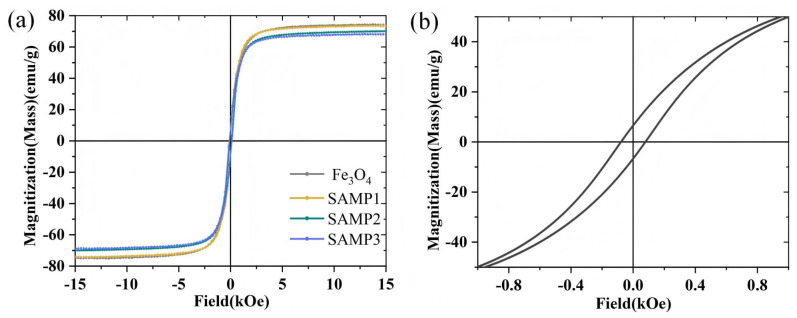
(**a**) Magnetic hysteresis loops of pure ${\mathrm{Fe}}_{3}O_{4}$ and three as-prepared samples. (**b**) The hysteresis loop of pure ${\mathrm{Fe}}_{3}O_{4}$ nanoparticles within the range of ±1 kOe.

As shown in Figure 4a, the pure ${\mathrm{Fe}}_{3}O_{4}$ nanoparticles (without rGO) exhibit remanence ($M_{r}$) and coercivity ($H_{c}$) values near zero, with no discernible hysteresis effect observed within the measurement range. The saturation magnetization ($M_{s}$) is relatively high, approximately 76 emu/g. As shown in the enlarged view of the low-magnetic-field region in Figure 4b, the pure ${\mathrm{Fe}}_{3}O_{4}$ sample exhibits very small coercivity and remanent magnetization, with values of only 79 Oe and 6.5 emu/g, respectively. Taking into account instrumental uncertainty, particle size distribution, and possible aggregation effects, these results indicate that the sample exhibits typical superparamagnetic behavior at room temperature. With the increasing content of rGO in the composites, the $M_{s}$ value gradually decreases to 68 emu/g. Nevertheless, the composites still maintain distinct superparamagnetic behavior [39]. The reduction in $M_{s}$ is attributed to the introduction of the non-magnetic rGO phase, which reduces the overall magnetic mass fraction in the composites.

### 3.3. Electromagnetic Parameters of Composites

**Figure 5:**
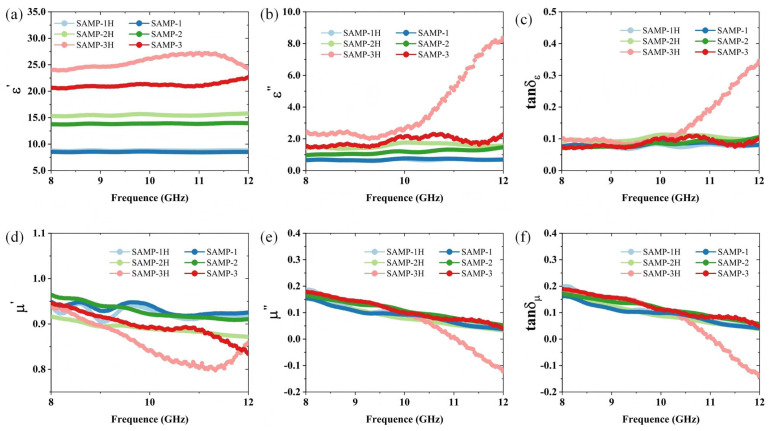
Electromagnetic parameters of ${\mathrm{Fe}}_{3}O_{4}$/rGO composites: (**a**) Real part of permittivity, (**b**) Imaginary part of permittivity, (**c**) Dielectric loss tangent, (**d**) Real part of permeability, (**e**) Imaginary part of permeability, (**f**) Magnetic loss tangent.

The complex permittivity and permeability of the prepared ${\mathrm{Fe}}_{3}O_{4}$/rGO composites were measured via the waveguide method in the frequency range of 8–12 GHz. Samples subjected to an external magnetic field are distinguished by the suffix “-H” in their labels. Figure 5a–f present the electromagnetic parameters of the ${\mathrm{Fe}}_{3}O_{4}$/rGO composites. As shown in Figure 5a,b, the increasing weight ratio of rGO leads to a simultaneous enhancement in the real part of the complex permittivity, which rises from approximately 8 (SAMP-1) to over 20 (SAMP-3). Meanwhile, a corresponding increase in the imaginary part is also observed. Consequently, the dielectric loss tangent ($\tan\delta_{\varepsilon }=\varepsilon^{\prime \prime }/\varepsilon^{\prime }$) is significantly improved (Figure 5c). Furthermore, the application of a magnetic field further enhances the dielectric loss capacity. This is attributed to the directional alignment of the rGO-based composites parallel to the incident electromagnetic waves, which creates a longer interaction path and results in stronger absorption [40]. These differences are more clearly illustrated by the Cole-Cole plots (Figure S3). The semicircles representing Debye relaxation become more prominent and increase in number with higher rGO content and the application of a magnetic field. This indicates that the abundant hetero-interfaces within the material trigger intensified interfacial polarization, thereby introducing additional relaxation processes [17,41].

Figure 5d–f illustrate the magnetic response characteristics of the ${\mathrm{Fe}}_{3}O_{4}$/rGO composites. It is observed that the real part of the complex permeability decreases as the rGO content increases. This decline is primarily ascribed to the increased volume fraction of the non-magnetic phase (rGO), which leads to a “dilution effect” that weakens the overall magnetic energy storage capacity of the material. In contrast, the imaginary part and the magnetic loss tangent ($\tan\delta_{\mu }=\mu^{\prime \prime }/\mu^{\prime }$) exhibit an upward trend across most of the frequency range. This phenomenon can be explained by the fact that the introduction of more rGO nanosheets promotes a more uniform and sparse dispersion of ${\mathrm{Fe}}_{3}O_{4}$, effectively preventing their agglomeration. The resulting increase in hetero-interfaces and the specific distribution of nanoparticles facilitate magnetic dissipation processes, such as natural resonance and superparamagnetic relaxation, thereby enhancing the magnetic loss capability [40].

Generally, the magnetic loss in magnetic materials primarily originates from hysteresis loss, domain wall resonance, eddy current loss, and natural resonance [4,42]. Among these, hysteresis loss and domain wall resonance typically occur under strong magnetic fields and at low frequencies; they are generally considered negligible in the microwave frequency range under the weak field conditions of a VNA measurement. Furthermore, domain wall resonance is exclusively associated with multi-domain materials. Given that the ${\mathrm{Fe}}_{3}O_{4}$ nanoparticles in this study have a particle size of approximately 10–20 nm—below the critical threshold for multi-domain structures—they can be regarded as single-domain particles [37]. Consequently, neither hysteresis nor domain wall resonance contributes significantly to the observed loss. Eddy current loss refers to the energy dissipation caused by induced currents within a conductive material under an alternating magnetic field. It is proportional to the square of the frequency and represents a major component of magnetic loss at high frequencies. To determine whether the magnetic loss is solely dominated by the eddy current effect, the eddy current coefficient ($C_{0}$) can be evaluated using the following relation [43].

(1) $$C_{0}=\frac{\mu^{\prime \prime }}{{\mu^{\prime }}^{2}f}=\frac{2}{3}\pi \mu_{0}D^{2}\sigma$$

where D represents the diameter of the magnetic particles, while $\mu^{\prime }$ and $\mu^{\prime \prime }$ denote the real and imaginary parts of the complex permeability of the material, respectively, $\mu_{0}$ is the permeability of free space, and σ is the material’s electrical conductivity. If the magnetic loss were solely attributed to eddy current loss, the $C_{0}$ value should remain constant across the investigated frequency range. By substituting the experimental permeability parameters into the aforementioned equation, the $C_{0}$ values for the 8–12 GHz range were calculated. As shown in Figure S4, the $C_{0}$ values of all samples decrease markedly with increasing frequency. For example, the $C_{0}$ value of SAMP-3 decreases from 0.025 at 8 GHz to 0.005 at 12 GHz, corresponding to a reduction of approximately 80%. A similar trend is also observed for the other samples. This trend clearly indicates that eddy current loss is not the dominant mechanism governing the magnetic dissipation. Considering that natural ferromagnetic resonance in magnetic nanomaterials typically occurs in the lower frequency range, it cannot fully account for the broadband response. According to the Kittel equation, the natural resonance relaxation expression for spherical magnetic particles is [44]:

(2) $$f_{r}=\frac{\gamma H_{k}}{2\pi }$$

where γ represents the gyromagnetic ratio, according to the cubic symmetry system, the equivalent magnetic crystal anisotropy field is $H_{k}=\frac{4\left|K_{1}\right|}{3\mu_{0}M_{s}}$ where the magnetic crystal anisotropy constant is $K_{1}=3.98\times 10^{2}\;$ J/m^3^, and the saturation magnetization is $M_{s}=3.63\times 10^{5}\;$A/m. The natural resonance frequency calculated from these parameters is 0.57 GHz. Furthermore, the broad peak distribution observed in the magnetic loss tangent is closely related to the wide particle size distribution of the superparamagnetic ${\mathrm{Fe}}_{3}O_{4}$. Consequently, it can be inferred that the broadband magnetic response at high frequencies primarily originates from superparamagnetic relaxation [44].

(3) $$f_{s}=f_{0}\exp\left(\frac{-KV}{k_{B}T}\right)$$

The relaxation frequency can be described by the Néel–Brown relaxation theory. In the related expression, $f_{0}$ represents the attempt frequency, which denotes the number of times per second that the magnetic moment attempts to overcome the energy barrier in the absence of thermal energy. Since $f_{0}$ is difficult to measure directly, a typical value ranging from $10^{9}$–$10^{11}$ Hz is generally adopted for ferrites [37]. *K* is the magnetocrystalline anisotropy constant, *V* is the size of the single-domain particle, $k_{B}$ is the Boltzmann constant, and *T* is the temperature. It is evident that the superparamagnetic relaxation frequency is strongly dependent on both particle size and temperature; specifically, it is inversely correlated with the particle volume (*V*) and positively correlated with the temperature (*T*). According to the Néel–Brown theory, the magnetic loss mechanism can be elucidated as follows: the emergence of magnetic loss peaks is primarily driven by the interaction between the Gilbert damping torque, generated during the reversal of magnetic moments under thermal fluctuations, and the externally applied alternating magnetic field [15].

### 3.4. Improved Absorption Properties of Composites

In the field of stealth technology, the minimum reflection loss (${\mathrm{RL}}_{\min}$), effective absorption bandwidth (EAB, RL<−10 dB), and matching thickness are the most critical parameters for evaluating performance [45].

(4) $$\mathrm{RL}\left(\mathrm{dB}\right)=20\mathrm{lg}\;\left|\frac{Z_{\mathrm{in}}-Z_{0}}{Z_{\mathrm{in}}+Z_{0}}\right|$$

(5) $$Z_{in}=Z_{0}\sqrt{\mu_{r}\varepsilon_{r}}\tanh\;\left[j\frac{2\pi fd}{c}\sqrt{\mu_{r}\varepsilon_{r}}\right]$$

**Figure 6:**
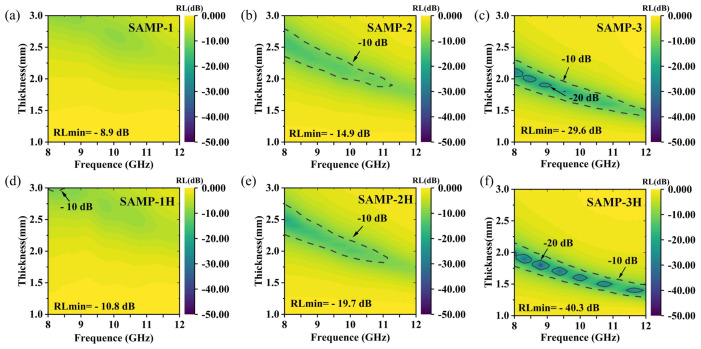
RL of ${\mathrm{Fe}}_{3}O_{4}$/rGO composites: (**a**) SAMP-1, (**b**) SAMP-2, (**c**) SAMP-3, (**d**) SAMP-1H, (**e**) SAMP-2H, and (**f**) SAMP-3H.

Based on transmission line theory, Figure 6 displays the calculated RL contour maps for composites with different rGO contents. By increasing the weight fraction of rGO, the microwave absorption properties of the composites were significantly enhanced. The ${\mathrm{RL}}_{\min}$ improved from −8.9 dB to −29.6 dB, while the EAB was simultaneously extended to cover the entire X-band (8–12 GHz). Furthermore, the application of an external magnetic field led to a distinct improvement in absorption performance across all doping ratios. Notably, when the rGO content reached 10 wt%, the material achieved full-band effective absorption (8–12 GHz) within a thickness range of 1–3 mm. Compared with the SAMP-3 sample prepared without an external magnetic field, the magnetically treated SAMP-3H sample exhibits superior microwave absorption performance. While maintaining effective absorption across the entire X-band, its minimum reflection loss is further enhanced from −29 dB to −40.3 dB. This result indicates that the magnetic-field-induced orientation significantly improves the electromagnetic wave attenuation capability of the composite.

**Figure 7:**
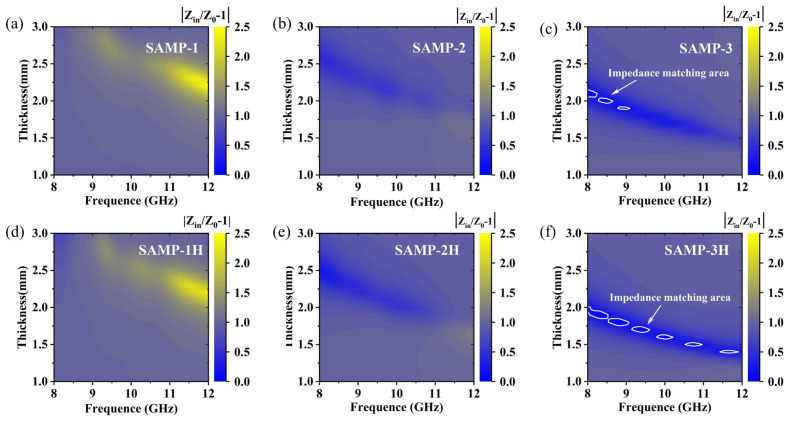
$\left|Z_{in}/Z_{0}-1\right|$ of ${\mathrm{Fe}}_{3}O_{4}$/rGO composites: (**a**) SAMP-1, (**b**) SAMP-2, (**c**) SAMP-3, (**d**) SAMP-1H, (**e**) SAMP-2H, and (**f**) SAMP-3H.

The superior electromagnetic wave attenuation capability of the material can be further elucidated by analyzing the impedance matching ($\left|Z_{in}/Z_{0}\right|$) and the attenuation constant (α) [46]. The impedance matching degree is characterized by the ratio of the material’s input impedance($Z_{in}$) to the impedance of free space($Z_{o}$), expressed as $\left|Z_{in}/Z_{0}\right|$. Generally, a $\left|Z_{in}/Z_{0}\right|$ value closer to 1 signifies that the material’s impedance is better matched to that of air, allowing incident electromagnetic waves to penetrate the interface more effectively rather than being reflected [47]. This facilitates stronger interaction between the waves and the material, leading to enhanced absorption and dissipation. As shown in Figure 7, $\left|Z_{in}/Z_{0}-1\right|$ is used to represent the degree of impedance matching. It is defined that $\left|Z_{in}/Z_{0}-1\right|<0.2$ indicates perfect impedance matching. By optimizing the rGO doping ratio, the dielectric loss and magnetic loss are effectively balanced, resulting in a significant enhancement of the impedance matching properties. Specifically, as shown in Figure 7c,f, when the rGO content reaches 10 wt%, the material achieves perfect matching at specific thicknesses and frequency bands. This phenomenon arises because rGO sheets oriented parallel to the direction of electromagnetic-wave propagation facilitate easier wave entry into the material compared to a disordered distribution. As shown in Figure S5, the α values of the composites increase with increasing rGO content, indicating enhanced electromagnetic wave attenuation capability within the materials. For example, the average α value increases from approximately 50 for SAMP-1 to approximately 90 for SAMP-3. In addition, within the frequency range of 8–12 GHz, SAMP-3 exhibits an average α value of approximately 90, whereas that of SAMP-3H increases to approximately 110, which is significantly higher than that of SAMP-3 prepared without an external magnetic field. These quantitative results further demonstrate that both the increased rGO content and the magnetic-field-induced orientation contribute to improving the electromagnetic attenuation capability of the composites. The increase in the attenuation constant (α) induced by the external magnetic field can be attributed to the fact that the parallel alignment of rGO sheets extends the interaction pathways between the material and incident electromagnetic waves, ultimately leading to enhanced absorption and increased energy dissipation.

Compared with the microwave absorption performance of recently reported representative magnetic carbon-based absorbers listed in Table 2, the magnetic-field-induced enhanced ${\mathrm{Fe}}_{3}O_{4}$/rGO composite proposed in this study achieves synergistic regulation of both composition and structure, exhibiting comprehensive advantages in microwave absorption, including a broad absorption bandwidth, a thin matching thickness, and strong reflection loss. Specifically, the minimum reflection loss reaches −40.3 dB at 8.79 GHz, and the effective absorption bandwidth (≤–10 dB) covers the entire 8–12 GHz range at a relatively thin matching thickness, outperforming most comparable materials.

**Figure 8:**
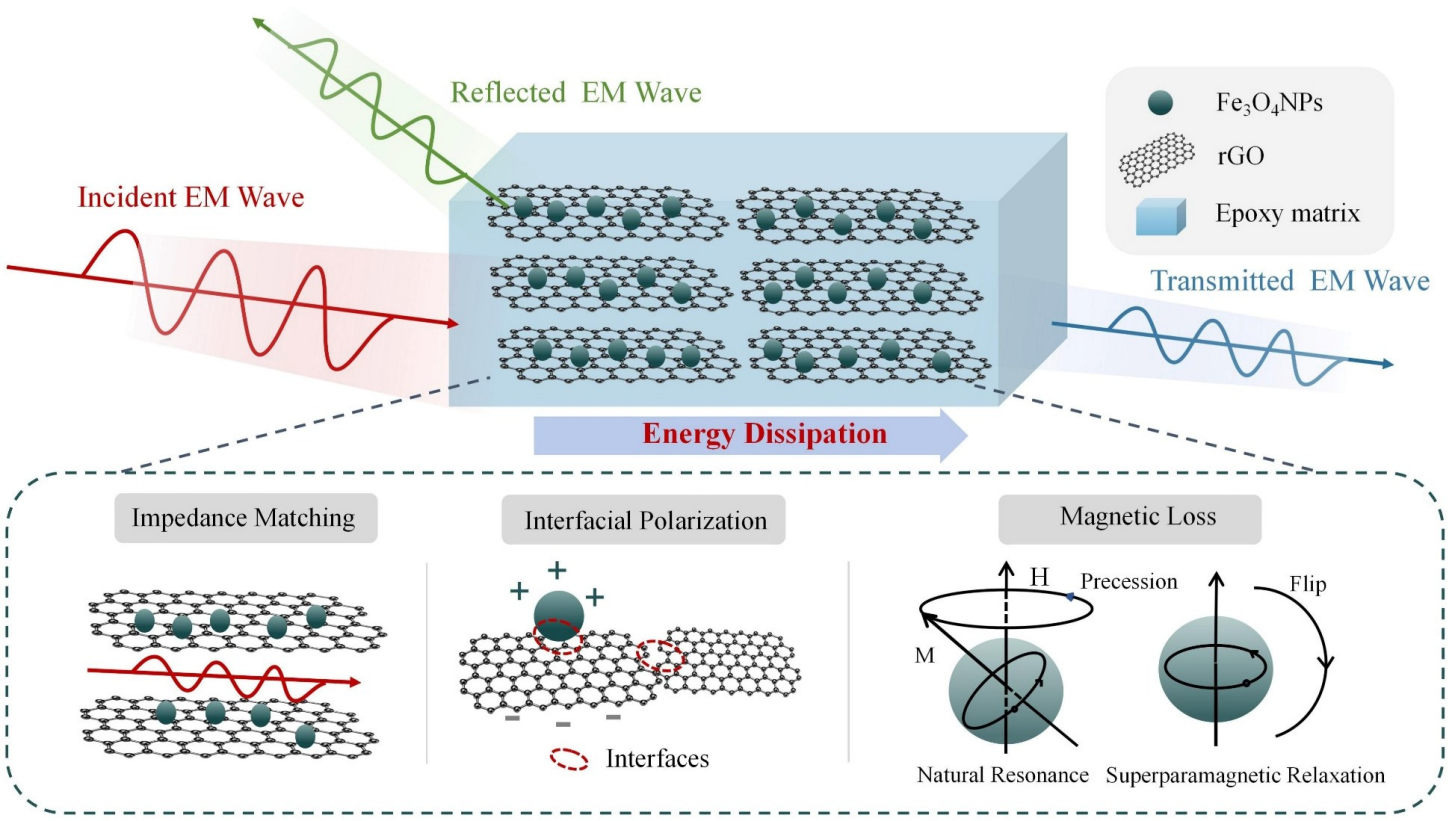
Schematic illustration of EMA mechanisms.

Finally, the microwave absorption mechanism is comprehensively elucidated in Figure 8. By fabricating the composite through the electrostatic adsorption of rGO and superparamagnetic ${\mathrm{Fe}}_{3}O_{4}$ nanoparticles, the material integrates magnetic loss dominated by superparamagnetic relaxation with interfacial polarization arising from the abundant heterogeneous interfaces. Complemented by magnetic-field-induced oriented structures that extend the attenuation paths of electromagnetic waves, the composite achieves maximized electromagnetic wave absorption performance.

## 4. Conclusions

In summary, a series of ${\mathrm{Fe}}_{3}O_{4}$/rGO composites were synthesized, and the influences of rGO doping ratios and external magnetic fields on their electromagnetic response were thoroughly investigated. Through the electrostatic adsorption method, ${\mathrm{Fe}}_{3}O_{4}$ nanoparticles were successfully anchored onto single-layer rGO sheets, while the oriented distribution of rGO within the material was achieved via an external magnetic field. Both experimental and calculated results demonstrate that the electromagnetic properties of the composites are highly enhanced by modulating the material ratios and magnetic field intensity. Notably, under an external magnetic field, the composite with a 10 wt% rGO doping ratio achieved effective absorption across the entire 8–12 GHz frequency range within a thickness of 1–3 mm, with a minimum reflection loss (${\mathrm{RL}}_{\min}$) of −40.3 dB. This work provides a novel approach for the micro-nano-scale modulation of composites and offers promising new insights for the engineering of advanced microwave absorbing materials.

## Figures and Tables

**Table 1:** Mass fractions of ${\mathrm{Fe}}_{3}O_{4}$/rGO in epoxy resin composites.

Sample Name	Filler Content (Mass Fraction)
SAMP-1	50 wt% ${\mathrm{Fe}}_{3}O_{4}$ NPs + 2.5 wt% rGO
SAMP-2	50 wt% ${\mathrm{Fe}}_{3}O_{4}$ NPs + 5 wt% rGO
SAMP-3	50 wt% ${\mathrm{Fe}}_{3}O_{4}$ NPs + 10 wt% rGO

**Table 2:** Microwave absorption performance of different electromagnetic wave absorbing materials.

Filler	RL_min_ (dB)	EAB (GHz)	Thickness (mm)	Refs.
${\mathrm{Zn}}_{0.8}{\mathrm{Ni}}_{0.2}{\mathrm{Fe}}_{2}O_{4}$	−18.95	3.1 (8.9–12)	2	[6]
${\mathrm{Fe}}_{3}O_{4}/\mathrm{Si}$	−29	2.4 (6.9–9.3)	2	[14]
${\mathrm{Fe}}_{3}O_{4}/{\mathrm{SiO}}_{2}$	−20.3	1.0 (5–16)	6	[15]
Ni@MWCNT/PS	−33	0.9 (2.6–3.5)	6	[21]
${\mathrm{Fe}}_{3}O_{4}$/MWCNT	−36	7.6 (5–12.6)	3	[25]
${\mathrm{Fe}}_{3}O_{4}$	−55	4.05 (3.15–7.2)	3.85	[35]
${\mathrm{BaFeCo}}_{0.6}O@{\mathrm{Fe}}_{3}O_{4}$	−23.8	2.8 (5.5–8)	3	[41]
${\mathrm{Fe}}_{3}O_{4}$/rGO	−40.3	4.0 (8–12)	1.8	This work

## Data Availability

The original contributions presented in this study are included in the article. Further inquiries can be directed to the corresponding author.

## Supplementary Materials

The following supporting information can be downloaded at https://www.mdpi.com/article/10.3390/mi17060754/s1. Figure S1: Zeta potentials of Fe_3_O_4_ and rGO. Figure S2: (a) Schematic of the experimental setup for curing composites under an external magnetic field; (b) Measurement of the magnetic field. Figure S3: The Cole-Cole curves of Fe_3_O_4_/rGO composites. Figure S4: C_0_ of Fe_3_O_4_/rGO composites. Figure S5: α of Fe_3_O_4_/rGO composites.
