# Supplementary material for: Magnetic-Field-Enhanced Microwave Absorption of Superparamagnetic Fe3O4/RGO Composites
Source: Micromachines (Basel). 2026 Jun 22;17(6):754. doi: 10.3390/mi17060754 (PMC13303179; doi:10.3390/mi17060754)
Supplement: Supplementary file 1 [file micromachines-17-00754-s001.zip › micromachines-4333311-supplementary.pdf]

## Supplementary Information

This Supplementary Information (SI) provides additional experimental details, images, and figure descriptions related to the main article. It specifically includes: (i) Zeta potentials of $Fe_3O_4$ and rGO as a function of pH values; (ii) the experimental apparatus, waveguide molds, and the magnetic field strengths measured by a Gaussmeter; and (iii) supplementary electromagnetic parameters of the $Fe_3O_4$/rGO composites, including Cole-Cole plots, the eddy current coefficient ($C_0$), and the attenuation constant ($\alpha$).

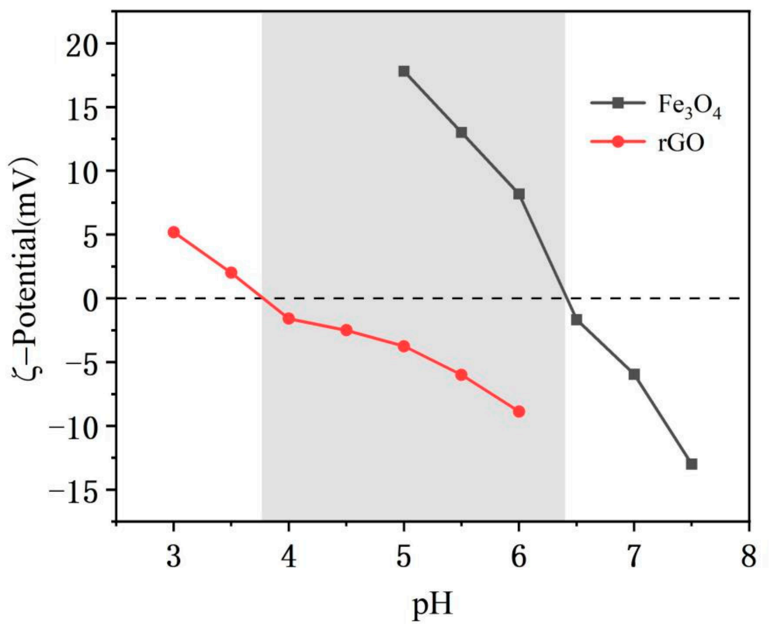


**Figure S1.** Zeta potentials of $Fe_3O_4$ and rGO.

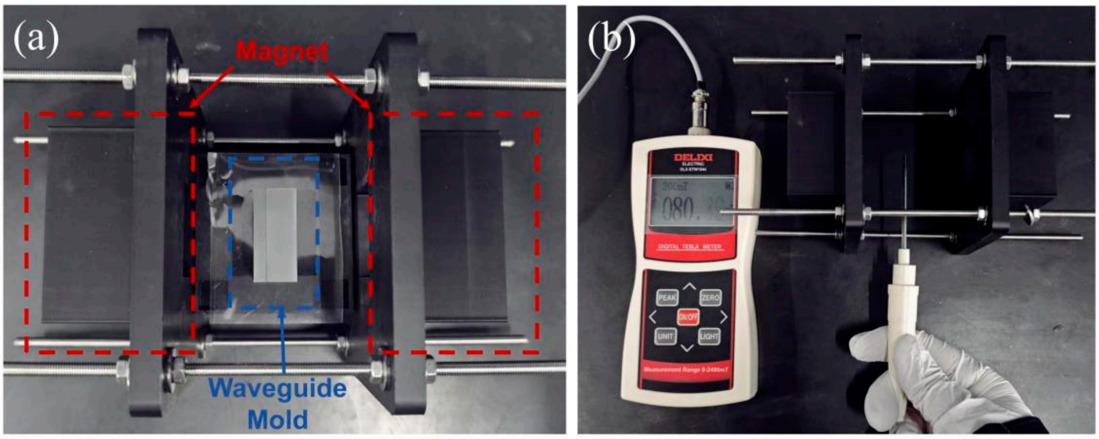


**Figure S2.** (a) Schematic of the experimental setup for curing composites under an external magnetic field; (b) Measurement of the magnetic field.

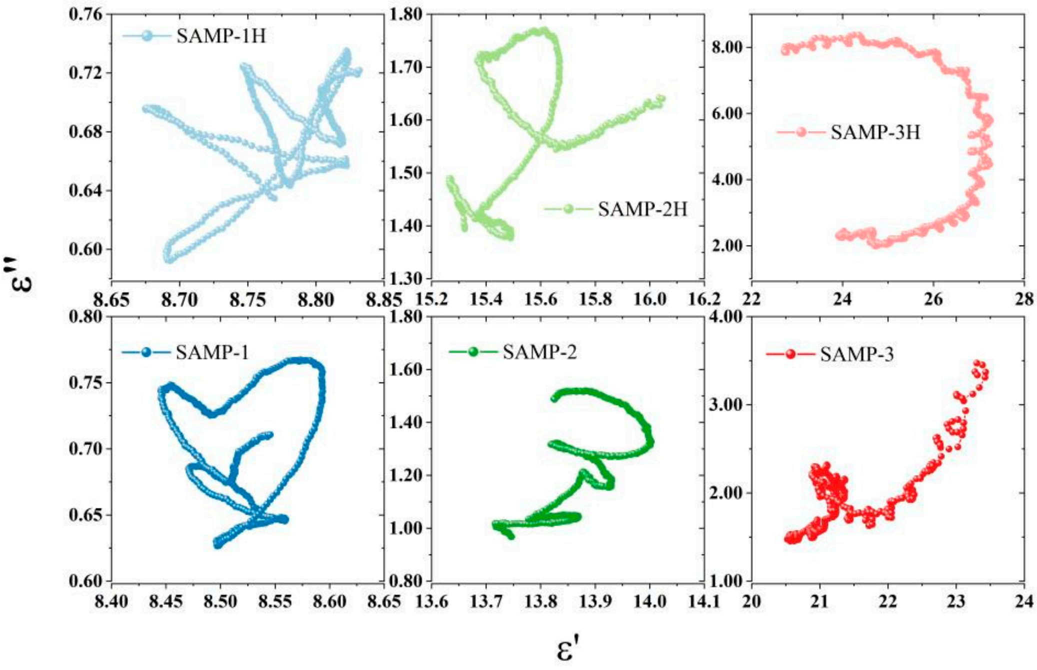


**Figure S3.** The Cole-Cole curves of $Fe_3O_4$/rGO composites.

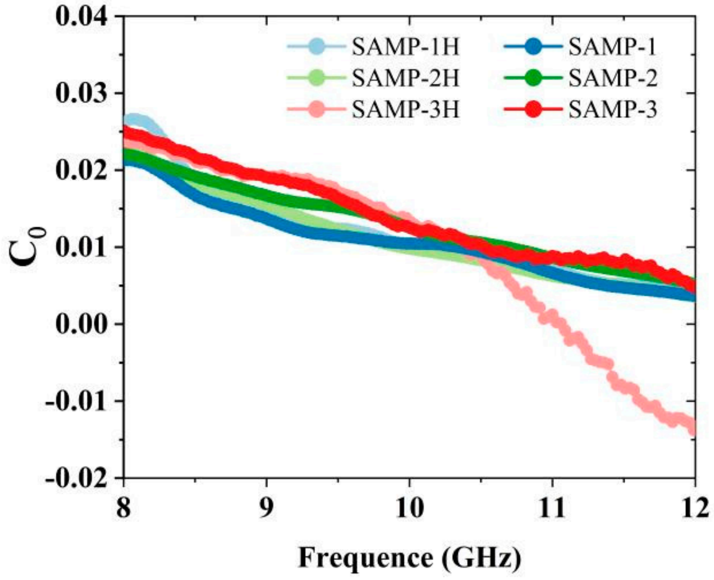


**Figure S4.** $C_0$ of $Fe_3O_4$/rGO composites.

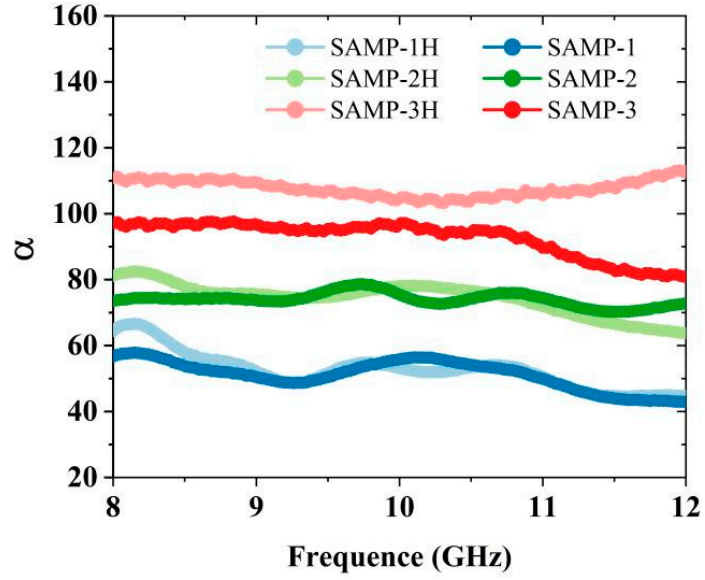


**Figure S5.** $\alpha$ of $Fe_3O_4$/rGO composites.
